# Prevalence of vision impairment, diabetic retinopathy and disability in adults 50+ in the occupied Palestinian territories

**DOI:** 10.1371/journal.pgph.0003613

**Published:** 2024-09-26

**Authors:** Nahed Mikki, Ian McCormick, Islay Mactaggart

**Affiliations:** 1 St. John of Jerusalem Eye Hospital Group, East Jerusalem; 2 International Centre for Eye Health, London School of Hygiene & Tropical Medicine, London, England; Duke University, UNITED STATES OF AMERICA

## Abstract

The Rapid Assessment of Avoidable Blindness methodology is a population-based survey of vision impairment among the population 50 and above, with optional modules on diabetes, diabetic retinopathy and disability. The first Rapid Assessment of Avoidable Blindness study in the occupied Palestinian territories (oPt) was conducted in 2008. Prevalence of blindness (50+) was 3.4%. 80% of blindness was avoidable. Between July 2018 and April 2019, we completed a nationally-representative follow up survey in oPt using the Rapid Assessment of Avoidable Blindness methodology including the optional modules. We tested distance visual acuity (presenting and pinhole) using a bespoke mobile data collection application. 4223 Palestinians aged 50 years and above were enumerated, of whom 3847 participated (response rate 91.1%). Prevalence of any vision impairment (presenting vision impairment <6/12 in the better seeing eye), blindness (<3/60), severe vision impairment (<6/60 but ≥3/60), moderate vision impairment (<6/18 but ≥6/60) and mild vision impairment (<6/12 but ≥6/18) were 25.8% (95% confidence interval [CI] 23.8–27.8%), 2.6% (1.9–3.2%), 1.4% (1.0–1.8%), 10.2% (9.1–11.2%) and 11.6% (10.3–12.8%), respectively. Avoidable causes of poor vision accounted for 82.4% of blindness, 83.3% of severe vision impairment, 82.0% of moderate vision impairment and 90.2% of mild vision impairment. Diabetes prevalence (reported or suspected based on random blood glucose ≥200 milligrams/decilitre) was 33.8% (32.1–35.5). Half of diabetes participants had diabetic retinopathy and/or maculopathy. Prevalence of disability (reported functional limitations) was 23.8% (21.0–26.5), and higher in women than men. The prevalence of vision impairment and blindness in oPt compared with 2008 was similar. Prevalence of diabetes, diabetic retinopathy and disability were all high, highlighting key areas for public health prioritization among older adults in oPt.

## Introduction

The decade 2021–2030 has been declared the Decade of Healthy Ageing by the United Nations, with an emphasis on enabling well-being in older age through developing and maintaining functional ability [1]. Vision impairment, diabetes and disability are all more common in older adults, and concerted efforts are required to achieve healthy ageing through provision of timely, quality services that maintain functioning and limit avoidable multimorbid functional decline [2, 3]

In 2020, the all-age prevalence of blindness and moderate or severe vision impairment in the North Africa and Middle East region was 0.7% (95% Confidence Interval [CI] 0.6–0.8) and 4.3% (3.9–4.7) respectively compared to global estimates of 0.5% (0.5–0.6) and 3.6 (3.2–3.9) from the same time [4]. There is strong evidence that vision and eye health affect quality of life, earnings and overall functioning [5–7]. Vision impairment and blindness are more common in low- and middle-income countries, among older people, and in rural communities [2]. Access to quality, affordable eye care fundamentally affects vision impairment prevalence, as 90% of all vision impairment is treatable or preventable through low-cost interventions [2].

The occupied Palestinian territory (oPt) comprises East Jerusalem, the West Bank and the Gaza Strip, with a total population of 4.7 million at the last census in 2017 [8]. 29.2% of the population was living below the poverty line, ranging from 13.9% in the West Bank to 53.0% in the Gaza Strip. The Gaza Strip is one of the most densely populated areas in the world and has been under complete blockade since 2007 [9]. The single largest provider of expert eye care services in the oPt is the St. John Eye Hospital Group, a charitable foundation that operates six hospitals and clinics in East Jerusalem, Gaza and the West Bank, in addition to three mobile outreach clinics in service of the most vulnerable population groups in isolated areas across the oPt to improve access to services among populations restricted by blockades [10]. In 2010, it was estimated that 68 ophthalmologists operated in the West Bank and 74 in Gaza but that the majority in the latter did not perform cataract surgery due to lack of training, with limited other secondary or tertiary eye health services available.

The Rapid Assessment of Avoidable Blindness (RAAB) is a population-based survey of blindness and vision impairment that samples the population 50 years and older, among whom vision impairment prevalence is highest [11]. RAAB captures key data on the coverage and quality of cataract services, with optional modules on diabetes and disability.

The first RAAB study in oPt was conducted in 2008 [12]. The prevalence of blindness in the population 50 years and older in this study was 3.4% (2.7–4.0%). Blindness prevalence was higher in women compared to men, and in Gaza compared with the West Bank [12]. The survey estimated that 80% of blindness was avoidable, with cataract the dominant cause of blindness and moderate or severe vision impairment, followed by uncorrected refractive error. Cataract surgical coverage (CSC) at the <3/60 surgical threshold was high– 86% of people who needed cataract surgery at this threshold had been operated. However, only 54.5% of cataract operated eyes obtained a post-operative visual acuity (VA) of ≥6/18.

Diabetes mellitus (DM) is the eighth leading cause of death and disability worldwide, with increasing prevalence globally and the highest age-standardised estimates in the North Africa and Middle East region (9.3%; 49.9 million people) [13]. Approximately 300,000 Palestinians (9.7% of the all-age population) were estimated to have diabetes in 2020, compared with a projected 17.7% by 2050. Diabetic retinopathy (DR) is a common complication of diabetes, and a leading cause of preventable blindness in this group [14].

Disability is described by the World Health Organization (WHO) as the interaction between a health condition or impairment, and contextual factors that can limit an individual’s equal participation in society [15]. 16.3% of the global population are estimated to have a disability, which is associated with lower access to healthcare, including eye health services, and poorer quality of life. The Palestinian Central Bureau of Statistics (PCBS) estimated that there were 93,000 Palestinians with disabilities comprising 2.1% of the population in 2017 [16].

### Rationale and aim of the study

RAAB surveys are usually conducted approximately every 10 years. Trends in the prevalence of blindness and vision impairment and service coverage are important for planning and monitoring services. The existing evidence on the prevalence of DR in oPt is limited to clinic-based populations. No national studies and no population-based studies of DR or disability have ever been conducted in oPt.

The aim of this survey was to assess the prevalence and causes of vision impairment, the prevalence of diabetes and diabetic retinopathy and the prevalence of disability in people aged 50 years and older.

Since our study, an escalated conflict between Palestinian armed groups–mainly Hamas–and the Israeli military has caused largescale destruction to Gaza. At the time of this paper’s publication in July 2024, this conflict is ongoing and has led to an estimated 38,000 deaths and 87,000 injuries, with over 75% of the population of Gaza estimated to have been internally displaced [17, 18]. We reflect on the potential impact of this on our findings in our discussion.

## Methodology

### Sample strategy and size

The survey was a nationally representative, cross-sectional, population-based survey in Gaza Strip and West Bank, conducted between 25^th^ July 2018 and 24^th^ April 2019. We used a two-stage cluster random sampling approach, following the standard RAAB protocol [19]. First, census enumeration areas were selected from a sampling frame as primary sampling units (clusters) with probability proportionate to size. Second, within each cluster, 35 people aged 50 years or older were randomly selected for examination in their homes via modified compact segment sampling. Based on an expected prevalence of blindness of 3.4% in individuals aged 50 years and older, desired relative precision of 20% (i.e. +/- 1.4%), confidence level 95%, non-response rate 10% and a design effect of 1.4 to account for clustering, a sample size of 4224 participants aged 50 years and older in 121 clusters of 35 was determined. The required sample size was calculated using the RAAB6 software [11, 12].

### Sampling frame and methodology

The sampling frame was a list of enumeration areas from the PCBS [8]. Enumeration Areas within the East Jerusalem walls were excluded from the survey because PCBS did not have any information on them. 69 clusters in the West Bank and 52 clusters in the Gaza Strip were selected from the list with probability proportionate to size. At the time of the survey, approximately 12% of the total Palestinian population in the oPt was over 50 years old [8], requiring segmentation of selected clusters into segments with a total population of 300 people to identify 35 people aged 50 years and older. One segment was then randomly selected. If the selected enumeration area did not include 35 people 50 years and older, the closest enumeration area was selected to complete the sample. If more than one enumeration area was close to the sample enumeration area, one enumeration area was randomly selected.

All persons aged 50 years and older, residing in a household within the selected segment for six months or more over the past year, were included in the survey. Visitors were excluded. The examination team sequentially visited households until 35 people 50 years and above were identified. If an eligible person was absent, the examination team returned to the household on the same day to examine the individual before leaving the area. For eligible individuals who could not be examined on the day, information on visual status was collected from relatives or neighbours. The survey team arranged to revisit at an appropriate time. If a house was locked, survey teams checked with neighbours whether any people of 50 years and above lived there. If this was the case, the basic information was recorded in a RAAB survey record and the house was revisited later. All unavailable, eligible participants were recorded as non-responders. Houses were skipped (no data recorded) if no information could be gathered from neighbours or if neighbours advised that the inhabitants were away for a long period (more than one night). The survey continued door-to-door route starting from a pre-agreed corner of the segment (e.g. North-East) until all the houses in that area were visited or until 35 people aged 50 years and above were included.

### Ophthalmic evaluation and data collection

The standard RAAB protocol was completed for each eligible person in the participant’s household, using a pilot version of RAAB7 –the latest iteration of the RAAB methodology which uses a bespoke mobile data collection application deployed on an Android device [19].

Participants were asked about any problems in their eyes and whether spectacles (distance and near) were used before having their distance VA tested using a validated mobile-based vision acuity test embedded within the survey app [20]. The classification of vision impairment used was in accordance with the International Classification of Diseases Version 11, as used by WHO [21]. Presenting VA (PVA, ie. with available correction, if worn) of 6/12 in the better eye was categorised as normal vision, mild vision impairment (VI) was categorized as <6/12 and ≥6/18; moderate VI as <6/18 and ≥6/60; severe VI as <6/18 and ≥3/60 and blindness as worse than 3/60.

All eyes with PVA <6/12 were re-tested with pinhole to estimate uncorrected refractive error. If the person wore spectacles, the pinhole was placed in front of the spectacles.

Lens status was assessed with a portable slit lamp for all participants without dilatation of the pupil. The lens was graded as: normal lens or minimal lens opacity, obvious lens opacity, absent lens (aphakia), intraocular implant (IOL) without posterior capsule opacification or IOL implant with posterior capsule opacification. If the lens could not be seen because of corneal scarring, Phthisis bulbi or other causes, ‘No view of lens’ was marked.

If VA improved to 6/12 or better with pinhole, then uncorrected refractive error was assigned as the cause of vision impairment. If VA did not improve to 6/12 or better and there was no lens or corneal abnormality, the pupil was dilated with short-acting mydriatic (tropicamide 0.5%) eye drops. The pupil was not dilated if very shallow chamber, obvious white cataract, large corneal opacity or occlusion pupillae were found. Once dilated, the lens (intraocular lens if present), posterior capsule and anterior vitreous were examined with a slit lamp in a semi-dark room by a licensed ophthalmologist.

The principal cause of vision impairment or blindness in the person was assessed based on WHO convention whereby the principal cause is attributed to the most treatable disorder. When there were multiple causes in the same eye or person, the cause that could be most easily treated or prevented was assigned the principal cause. Avoidable principal causes were further categorized as treatable or preventable. Treatable causes included uncorrected refractive error, cataract and aphakia. Preventable causes included diabetic retinopathy, glaucoma, cataract surgical complications, trachomatous corneal opacity, non-trachomatous corneal opacity and phthisis.

All enrolled participants were invited to participate in the diabetes and diabetic retinopathy module. If participants consented, a random blood glucose (RBG) test was conducted by a trained nurse. Used needles were collected in a safety box and disposed of at the institute in charge of the research project. Participants were classified as having ‘known’ DM if they had been previously diagnosed by a health professional, or ‘suspect’ DM if their random blood glucose (RBG) was 200 mg/dL or greater on the day of data collection. Dilated fundus examination and Scottish DR grading of old and newly diagnosed diabetes participants were performed by an ophthalmologist using a portable slit lamp in a darkened room [22].

Disability was assessed using the Washington Group Short Set, a six-question reported functional limitations module endorsed for capturing population disability data by the United Nations [23]. Participants reporting “a lot of difficulty” or worse in any of the six domains (seeing, hearing, mobility, communication, cognition and self-care) were considered to have a disability.

Three teams were trained to complete the data collection. Two were assigned to the West Bank and one was assigned to Gaza. Each team consisted of one experienced ophthalmologist, one nurse and one driver. All teams received one-week training in theoretical and practical aspects of the RAAB methodology by a qualified RAAB trainer (IZM) including an inter-observer variation (IOV) exercise. A pre-specified kappa statistic minimum score of 0.6 was required for each of VA assessment, lens exam and assigned cause of blindness before the survey could begin. The training program included a pilot visit to a study location with the RAAB trainer.

Data entry, IOV testing and standardized data analysis were conducted using RAAB7 software (London School of Hygiene & Tropical Medicine, UK) which was used for the first time in oPt. All analyses in this report were done using the RAAB7 code available at https://github.com/raabteam/raab7-analysis.

Cataract Surgical Coverage (CSC) and effective Cataract Surgical Coverage (eCSC) were calculated in line with WHO recommendations as described in Box 1 [24]. The Relative Quality Gap was calculated as (CSC-eCSC)/CSC in line with McCormick et al. (2022).

**Calculation for Cataract Surgical Coverage (CSC)**

$$CSC=\frac{x+y}{x+y+z}$$

where *x* is individuals with unilateral operated cataract (regardless of visual acuity in the operated eye) and vision impairment (using Best Corrected Visual Acuity, BCVA*) in the other eye, *y* is individuals with bilateral operated cataract (regardless of visual acuity in the operated eyes), and *z* is individuals with vision impairment (using BCVA*) in both eyes with cataract as the main cause of vision impairment in one or both eyes.†

**Calculation for Effective Cataract Surgical Coverage (eCSC)**

$$eCSC=\frac{a+b}{x+y+z}$$

Where *a* is individuals with unilateral operated cataract attaining a specified threshold of postoperative presenting visual acuity in the operated eye, who have vision impairment (using BCVA*) in the other eye and *b* is individuals with bilateral operated cataract attaining a specified threshold of postoperative presenting visual acuity in at least one eye.†

*According to the cataract surgical threshold used in the estimate (<6/12, <6/18, <6/60, or <3/60). †Cases of couched eyes are excluded from the count of x, y, a, and b.

Reproduced from McCormick, I., Butcher, R., Evans, J. R., Mactaggart, I. Z., Limburg, H., Jolley, E.,… & Zhang, X. J. (2022). Effective cataract surgical coverage in adults aged 50 years and older: estimates from population-based surveys in 55 countries. *The Lancet Global Health*, *10*(12), e1744-e1753.

Estimates of the number of cases of blindness, severe vision impairment, moderate vision impairment and early vision impairment in oPt were obtained by extrapolating the age-sex-specific prevalence estimates to the age-sex structure of the Palestinian population.

### Ethical approval

Ethical approval was obtained from St John Eye Hospital in East Jerusalem Internal Ethics Review Board and London School of Hygiene & Tropical Medicine Observational Ethics Committee. Approval was also obtained from the Palestinian Ministry of Local governance, head of municipalities, camps and villages.

Witnessed oral informed consent was obtained from all participants, in keeping with standard practices for oPt. The purpose and the procedures of the study were described, and participants were informed that their participation was voluntary, they could withdraw at any time or refuse any part of the study and that all collected information would be kept confidential for scientific purposes only. All participants received feedback about their examination and participants with abnormal findings were provided counseling and offered a free consultation at the research institute’s facilities.

### Inclusivity in global research

Additional information regarding the ethical, cultural, and scientific considerations specific to inclusivity in global research is included in the Supporting Information (S1 Checklist)

## Results

The survey was conducted across the Occupied Palestinian Territories (oPt) between July 2018 and April 2019. We enumerated 4,223 Palestinians aged 50 years and above, of whom 3,847 (91.1%) participated in the study (Table 1). Among non-responders, 4.2% were not available at the time of the survey, 3.8% were not capable of participating in the survey and 0.9% refused to participate. Non-response was 12.5% among men and 7.5% among women, and 14.2% in the West Bank compared with 1.9% in Gaza.

**Table 1:** Sample characteristics.

	Sample																		Survey Area (Census)					
	oPt						West Bank						Gaza											
	Female		Male		Total		Female		Male		Total		Female		Male		Total		Female		Male		Total	
Age Group	n	%	n	%	n	%	n	%	n	%	n	%	n	%	n	%	n	%	n	%	n	%	n	%
50–59	954	46.6	812	45.1	1766	45.9	530	48.1	473	49.2	1003	48.6	424	45.0	339	40.4	763	42.8	135358	52.2	144685	56.9	280043	54.6
60–69	638	31.2	618	34.3	1256	32.6	313	28.4	305	31.7	618	29.9	325	34.5	313	37.3	638	35.8	70781	27.3	70227	27.6	141008	27.5
70–79	324	15.8	264	14.7	588	15.3	187	17.0	129	13.4	316	15.3	137	14.5	135	16.1	272	15.3	35354	13.6	27910	11.0	63264	12.3
80+	130	6.4	107	5.9	237	6.2	73	6.6	55	5.7	128	6.2	57	6.0	52	6.2	109	6.1	17583	6.8	11407	4.5	28990	5.6
Total	2046	100	1801	100	3847	100	1103	100	962	100	2065	100	943	100	839	100	1782	100	259076	100	254229	100	513305	100

Compared with the 2017 census age-sex estimates, men and women were under-represented in the 50 to 59 age group. Men were over-represented in all other age groups and women were over-represented in the 60–69 and 70–79 groups. Census age- and sex- weighted estimates were calculated to adjust for discrepancies between the sample and the population.

Table 2 presents age-sex adjusted prevalence estimates of vision impairment (VI) and blindness separately for the West Bank and Gaza, and combined. The prevalence of any VI (PVA>6/12) in people 50+ across the oPt was 25.8% (95% CI 23.8–27.8%). The overall prevalence of blindness was 2.6% (1.9–3.2%), severe VI was 1.4% (1.0–1.8), moderate VI was 10.2% (9.1–11.2) and mild VI was 11.6% (10.3–12.8). Vision impairment was significantly higher in women than men overall (30.5%, 27.9–33.1 vs. 21.0%, 18.7–23.3), and for each impairment category except severe VI, for which estimates were similar by sex.

**Table 2:** Census age-sex adjusted prevalence of blindness, severe, moderate and mild vision impairment.

	oPt (n = 3848)						West Bank (n = 2065)						Gaza (n = 1783)					
	Female (n = 2047)		Male (n = 1801)		Total (n = 3848)		Female (n = 1103)		Male (n = 962)		Total (n = 2065)		Female (n = 944)		Male (n = 839)		Total (n = 1783)	
VI Level	%	% (95% CI)	%	% (95% CI)	%	% (95% CI)	%	% (95% CI)	%	% (95% CI)	%	% (95% CI)	%	% (95% CI)	%	% (95% CI)	%	% (95% CI)
Blind	3.6	2.6–4.6	1.5	0.9–2.2	2.6	1.9–3.2	2.5	1.6–3.4	0.9	0.3–1.4	1.7	1.1–2.3	4.8	3.1–6.5	2.1	0.9–3.3	3.5	2.4–4.6
Severe	1.3	0.9–1.8	1.5	0.9–2.0	1.4	1.0–1.8	1.3	0.7–2.0	1.6	0.8–2.4	1.5	0.9–2.0	1.3	0.6–2.0	1.4	0.6–2.1	1.3	0.8–1.9
Moderate	12.1	10.7–13.6	8.3	6.8–9.8	10.2	9.1–11.2	10.4	8.6–12.3	7.3	5.4–9.2	8.9	7.5–10.3	14.0	11.8–16.2	9.5	7.2–11.8	11.8	10.2–13.4
Mild	13.5	11.7–15.2	9.7	8.0–11.3	11.6	10.3–12.8	10.3	8.3–12.4	7.1	5.4–8.8	10.3	8.9–11.8	17.2	14.7–19.7	12.6	10.0–15.1	14.9	13.3–16.5
Any VI	30.5	27.9–33.1	21.0	18.7–23.3	25.8	23.8–27.8	24.6	21.9–27.2	16.8	14.3–19.3	20.7	18.7–22.7	37.3	33.6–41.1	25.5	22.3–28.7	31.5	28.7–34.3

VI = Vision Impairment

Extrapolating the survey estimates to the age and sex distribution of the oPt from the updated census data suggests that in the ≥50 years age group, there are an estimated 13,182 blind people, 7,214 severely visually impaired people, 52,523 moderately visually impaired and 59,489 people with mild vision impairment.

The prevalence of any VI was significantly higher in Gaza (31.5%, 28.7–34.3) than in the West Bank (20.7%, 18.7–22.7). There were no significant differences in the prevalence of severe VI and moderate VI between the West Bank and Gaza, but the prevalence of blindness (3.5%, 2.4–4.6 vs 1.7%, 1.1–2.3) and mild VI was higher in Gaza (14.9%, 13.3–16.5 vs. 10.3%, 8.9–11.8). The prevalence of any VI was significantly higher among women compared with men in both the West Bank and Gaza (West Bank: 24.6%, 21.9–27.2 vs 16.8%, 14.3–19.3; Gaza: 37.3%, 33.6–41.1 vs. 25.5%, 22.3–28.7). The prevalence of blindness was also significantly higher among women compared with men in both the West Bank and Gaza (West Bank: 2.5%, 1.6–3.4 vs. 1.7%, 1.1–2.3; Gaza: 4.8%, 3.1–6.5 vs. 2.1%, 0.9–3.3), but there were no differences by sex for severe, moderate or mild VI in either setting.

Avoidable causes of poor vision (untreated cataract, diabetic retinopathy, uncorrected refractive error, glaucoma, cataract surgical complications, corneal scar and phthisis) accounted for 82.4% of the total amount of blindness, 83.3% of severe VI, 82.0% of MVI and 90.2% of mild VI in oPt (Table 3). Cataract was the dominant cause of blindness (38.0%), severe visual impairment (45.0%) and moderate visual impairment (40.0%). Diabetic retinopathy was the second leading cause of blindness (24.1%), severe VI (31.7%) and moderate VI (17.8%) and the third cause of mild VI (14.4%). Uncorrected refractive error was the main cause of mild VI (45.2%).

**Table 3:** Proportion of vision impairment by cause in West Bank, Gaza and across the oPt.

	oPt								West Bank								Gaza							
	Blind		Severe		Moderate		Mild		Blind		Severe		Moderate		Mild		Blind		Severe		Moderate		Mild	
	n	%	n	%	n	%	n	%	n	%	n	%	n	%	n	%	n	%	n	%	n	%	n	%
1. Uncorrected refractive error	0	0	1	1.7	66	15.2	216	45.2	0	0	1	3.1	51	25.8	136	70.8	0	0	0	0	15	6.4	80	28.0
2. Uncorrected aphakia	0	0	0	0	0	0	1	0.2	0	0	0	0	0	0	1	0.5	0	0	0	0	0	0	0	0
3. Untreated cataract	41	38.0	27	45.0	173	40.0	114	23.8	19	51.4	19	59.4	84	42.4	28	14.6	22	31.0	8	28.6	89	37.9	86	30.1
4. Cataract surgical complications^1^	6	5.6	0	0	20	4.6	22	4.6	3	8.1	0	0	11	5.6	4	2.1	3	4.2	0	0	9	3.8	18	6.3
5. Trachomatous corneal opacity	1	0.9	0	0	0	0	0	0	0	0	0	0	0	0	0	0	1	1.4	0	0	0	0	0	0
6. Other corneal opacity	4	3.7	2	3.3	9	2.1	3	0.6	2	5.4	2	6.2	2	1.0	1	0.5	2	2.8	0	0	7	3.0	2	0.7
7. Phthisis	4	3.7	0	0	0	0	0	0	0	0	0	0	0	0	0	0	4	5.6	0	0	0	0	0	0
8. Glaucoma	7	6.5	1	1.7	11	2.5	6	1.3	2	5.4	0	0	6	3.0	0	0	5	7.0	1	3.6	5	2.1	6	2.1
9. Diabetic retinopathy	26	24.1	19	31.7	77	17.8	69	14.4	3	8.1	4	12.5	15	7.6	10	5.2	23	32.4	15	53.6	62	26.4	59	20.6
10. Age-related macular degeneration	8	7.4	3	5.0	21	4.8	18	3.8	2	5.4	1	3.1	3	1.5	3	1.6	6	8.5	2	7.1	18	7.7	15	5.2
11. Other posterior segment disease	7	6.5	5	8.3	38	8.8	25	5.2	3	8.1	4	12.5	17	8.6	7	3.6	4	5.6	1	3.6	21	8.9	18	6.3
12. Other globe or Central Nervous System abnormalities	4	3.7	2	3.3	18	4.1	4	0.8	3	8.1	1	3.1	9	4.5	2	1.0	1	1.4	1	3.6	9	3.8	2	0.7
Total	108	100	60	100	434	100	478	100	37	100	32	100	198	100	192	100	71	100	28	100	235	100	286	100

^1^Categorised as evidence that a cataract surgical procedure has led to a vision-impairing condition e.g. retinal detachment, or obvious complications from surgery visible with torch e.g. peaked pupil

Avoidable causes of poor vision in the West Bank accounted for 78.4%, 81.3%, 85.4% and 93.8% of blindness, severe VI, moderate VI and mild VI, respectively. In Gaza, avoidable causes accounted for 84.5%, 85.7%, 79.6% and 87.8% of blindness, severe VI, moderate VI and mild VI, respectively. The main cause of blindness and severe VI in the West Bank was untreated cataract (51.4% and 59.4% of causes respectively) versus diabetic retinopathy (32.4% and 53.6%) in Gaza. The main cause of moderate VI in both the West Bank and Gaza was untreated cataract, and the main cause of mild VI was uncorrected refractive error in the West Bank (70.8%) and untreated cataract (30.1%) in Gaza. Commonly reported barriers to accessing cataract surgery among participants with bilateral cataract were fear (25.9%), participant unaware treatment was possible (22.2%) and surgery denied by provider (20.4%).

At the 6/60 threshold, cataract surgical coverage (CSC) was high overall with 89.0% (95% CI 85.7–92.4) of people requiring surgery having received surgery (Table 4). This was higher in men (92.2%, 88.3–96.1) than in women (86.7%, 82.1–91.3). At the VA<6/18 and VA<6/12 thresholds, 74.4% (70.2–78.5) and 61.0% (57.1–64.9) of those needing surgery had received it, respectively, with no differences by sex. At the 6/60 threshold, less than half of those operated had achieved a post-operative VA of 6/12 (eCSC 47.5%, 42.3–52.8), decreasing to 37.6% (33.1–42.1) at the VA<6/18 threshold and 30.4% (26.7–34.1) at the VA<6/12 threshold respectively. CSC was similar in West Bank compared to Gaza, but eCSC was lower in Gaza leading to higher relative quality gaps at all thresholds.

**Table 4:** Census age-sex adjusted cataract surgical coverage and effective cataract surgical coverage by cataract surgical threshold.

	Cataract Surgical Threshold	Indicator	Women		Men		Total		
			Adj %	95% CI	Adj %	95% CI	Adj %	95% CI	Relative Quality Gap
oPt	Less than 6/60	CSC	86.7	82.1–91.3	92.2	88.3–96.1	89.0	85.7–92.4	
		eCSC	41.7	34.6–48.8	55.4	48.3–62.4	47.5	42.3–52.8	46.6
	Less than 6/18	CSC	71.8	66.9–76.6	78.3	72.0–84.5	74.4	70.2–78.5	
		eCSC	32.4	26.9–38.0	45.3	38.6–52.0	37.6	33.1–42.1	49.4
	Less than 6/12	CSC	58.2	53.7–62.7	65.2	59.3–71.0	61.0	57.1–64.9	
		eCSC	26.0	21.7–30.2	37.2	31.7–42.6	30.4	26.7–34.1	50.2
West Bank	Less than 6/60	CSC	86.0	78.2–93.7	86.0	78.1–93.9	86.0	80.1–91.9	
		eCSC	52.4	41.0–63.8	51.1	40.2–61.9	51.8	43.1–60.5	39.8
	Less than 6/18	CSC	67.6	60.3–74.9	68.4	56.2–80.6	67.9	61.0–74.8	
		eCSC	35.7	27.1–44.2	39.7	29.6–49.9	37.2	30.1–44.4	45.2
	Less than 6/12	CSC	54.9	48.2–61.5	61.3	50.0–72.6	57.2	50.8–63.6	
		eCSC	28.9	22.1–35.7	35.8	26.6–45.1	31.4	25.2–37.7	45.0
Gaza	Less than 6/60	CSC	87.3	81.5–93.1	96.6	93.1–100.0	91.2	87.3–95.0	
		eCSC	34.6	26.2–43.1	59.8	50.6–69.0	45.0	38.6–51.5	50.6
	Less than 6/18	CSC	75.6	69.1–82.0	84.9	79.4–90.3	79.4	75.0–83.7	
		eCSC	29.8	22.8–36.8	50.3	41.6–59.0	38.2	32.4–43.9	51.9
	Less than 6/12	CSC	60.9	54.8–67.0	67.0	60.6–73.4	63.4	58.8–68.0	
		eCSC	23.5	18.2–28.8	38.5	31.8–45.3	29.7	25.2–34.3	53.1

CSC = Cataract Surgical Coverage, defined as number of people with operated cataract as proportion of all people operated or still requiring surgery; eCSC = effective cataract surgical coverage defined as number of people with operated cataract and post operative visual acuity of 6/12 or better, as proportion of all people operated or still requiring surgery. Relative Quality Gap defined as (CSC-eCSC)/CSC

Among enrolled participants, 3778 (89.5%) consented to having their diabetes status assessed. Of these, 951 participants (74.4% of those identified to have known or suspected diabetes) consented to dilated examination. To account for this lower response rate, crude sample prevalence estimates are provided for these outputs in Table 5. Overall diabetes prevalence across oPt was 33.8% (32.1–35.5), and was significantly higher among women (36.8%, 34.2–39.3) than men (30.5%, 28.4–32.6), Table 5. Diabetes prevalence in Gaza was higher with borderline significance than in the West Bank (36.3%, 34.0–38.5 vs. 31.7%, 29.3–34.1) and higher in women compared with men in Gaza, but similar by sex in the West Bank.

**Table 5:** Sample prevalence of diabetes and diabetic retinopathy.

		Diabetes^1^	Diabetic Retinopathy^2^	
		Known or suspected diabetes	Any Diabetic Retinopathy	Sight Threatening Diabetic Retinopathy
oPt				
Female	n	737	260	102
	% (95% CI)	36.8 (34.2–39.3)	46.7 (41.2–52.2)	18.3 (14.5–22.1)
Male	n	541	194	73
	% (95% CI)	30.5 (28.4–32.6)	49.2 (43.8–54.7)	18.5 (14.6–22.5)
Total	n	1278	454	175
	% (95% CI)	33.8 (32.1–35.5)	47.7 (43.2–52.3)	18.4 (15.6–21.2)
West Bank				
Female	n	368	67	14
	% (95% CI)	34.2 (31.0–37.5)	28.4 (22.1–34.7)	5.9 (2.7–9.2)
Male	n	272	57	20
	% (95% CI)	28.8 (25.7–31.9)	33.7 (26.7–40.7)	11.8 (6.7–17.0)
Total	n	640	124	34
	% (95% CI)	31.7 (29.3–34.1)	30.6 (25.2–36.0)	8.4 (5.5–11.3)
Gaza				
Female	n	369	193	88
	% (95% CI)	39.7 (35.9–43.5)	60.1 (54.2–66.1)	27.4 (22.7–32.1)
Male	n	269	137	53
	% (95% CI)	32.5 (29.7–35.2)	60.9 (54.2–67.6)	23.6 (18.2–28.9)
Total	n	638	330	141
	% (95% CI)	36.3 (34.0–38.5)	60.4 (55.5–65.4)	25.8 (22.5–29.1)

^1^ Among enrolled participants who consented to having their diabetes status assessed (n = 3778, 89.5% of enrolled participants)

^2^Among known or suspected diabetics who consented dilated examination (n = 951, 74.4% of participants with known or suspected diabetes); Diabetic retinopathy (DR) categorised as per Scottish Grading System as follows: Any DR at least R1 or M1; Sight Threatening DR (STDR) R4 and/or M2; No DR R0 and M0

Only 539 (46.3%) of known diabetes patients had controlled blood glucose (RBG<200). More than 40% of people with known diabetes either had never had an eye check-up or had not been checked in the two years prior to the survey.

Across oPt, approximately half (47.7%, 43.2–52.3) of all diabetes participants had diabetic retinopathy and/or maculopathy (DR). This increased to 60.4% (55.5–65.4) in Gaza, compared with 30.6% (25.2–36.0) in the West Bank and was similar by sex overall and in both zones.

Any retinopathy and /or maculopathy was higher among older age groups. Prevalence of blindness was 3.4% (2.4–4.3) in diabetes participants compared to 2.5% (1.8–3.2) among non-diabetes patients; however, the difference was not significant.

The census age-sex adjusted prevalence of disability in oPt was 23.8% (21.0–26.5), and higher in women (28.5%, 25.0–32.1) compared with men (18.9%, 16.2–21.5), Table 6. Disability prevalence was similar in the West Bank (25.2%, 20.5–30.0) compared with Gaza (21.4%, 18.9–24.0), and higher in women compared with men in both zones (West Bank: 31.0%, 25.1–36.9 vs. 19.4%, 15.1–23.7; Gaza: 25.3%, 21.9–28.7 vs. 17.5%, 14.6–20.4). The prevalence of non-vision related disability was 18.1% (15.3–21.0) overall, and higher in the West Bank compared with Gaza (22.7%, 18.0–27.4 vs. 12.3%, 9.8–14.9). The prevalence of non-vision related disability was also higher in women compared with men in the oPt overall (22.6%, 18.9–26.3 vs 13.6%, 11.0–16.2), the West Bank (28.2%, 22.2–34.2 vs. 17.1%, 13.1–21.2) and Gaza (15.8%, 12.5–19.0 vs. 12.3%, 9.8–14.9).

**Table 6:** Census age-sex adjusted prevalence of disability.

		Any disability	Any non-vision related disability
oPt			
Female	n	601	476
	% (95% CI)	28.5 (25.0–32.1)	22.6 (18.9–26.3)
Male	n	372	271
	% (95% CI)	18.9 (16.2–21.5)	13.6 (11.0–16.2)
Total	n	973	747
	% (95% CI)	23.8 (21.0–26.5)	18.1 (15.3–21.0)
West Bank			
Female	n	348	317
	% (95% CI)	31.0 (25.1–36.9)	28.2 (22.2–34.2)
Male	n	194	173
	% (95% CI)	19.4 (15.1–23.7)	17.1 (13.1–21.2)
Total	n	542	490
	% (95% CI)	25.2 (20.5–30.0)	22.7 (18.0–27.4)
Gaza			
Female	n	253	159
	% (95% CI)	25.3 (21.9–28.7)	15.8 (12.5–19.0)
Male	n	178	98
	% (95% CI)	17.5 (14.6–20.4)	8.8 (5.9–11.7)
Total	n	431	257
	% (95% CI)	21.4 (18.9–24.0)	12.3 (9.8–14.9)

Disability categorised as per standard Washington Group definition as any domain reported “a lot of difficulty” or “cannot do”; Any non-seeing disability categorised as above, minus the vision domain

## Discussion

In 2018–2019 we conducted a follow up RAAB in oPt to compare estimates with the RAAB in 2008, and included additional data collection to capture the first population-based estimates of disability in the population 50+ years. This survey was the first ever pilot test of the current iteration of the RAAB survey methodology–RAAB7–that used a bespoke Android app to sync data to a cloud-based server and centralized survey management platform.

The age-sex weighted prevalence of blindness was 2.6% (1.9–3.2%), severe VI was 1.4% (1.0–1.8), moderate VI was 10.2% (9.1–11.2) and mild VI was 11.6% (10.3–12.8). Vision impairment was generally higher in women compared with men, and Gaza compared with the West Bank. Over 80% of VI was due to avoidable causes, with cataract and diabetic retinopathy the dominant causes of blindness and severe VI. Uncorrected refractive error was the main cause of mild VI overall and in the West Bank, second to cataract in Gaza. Both cataract surgical coverage (CSC) and effective CSC were high at the <6/60 surgical threshold, with both decreasing as the cataract surgical threshold was lowered. The prevalence of diabetes (33.8%), DR (47.7% among diabetic participants) and disability (23.8%) were all high, with both diabetes and disability more common in women than men.

The prevalence of severe vision impairment in Gaza was slightly lower in our study compared to the 2008 oPt RAAB (1.3% [95% CI 0.8–1.9] vs. 3.2% [2.1–4.2%]); however, the prevalence of all other vision impairment categories in oPt overall, and for West Bank and Gaza separately, were statistically similar to estimates from ten years prior [12]. Despite increasing eye health service provision, this may relate to a rapidly growing and ageing population–the number of Palestinians aged 50 years and above has increased 54% between 2008 and 2017, potentially doubling the need for services in this age group [8, 25]. It may also highlight the growing diabetes and consequently diabetic retinopathy epidemic in the country, associated with changing lifestyles and nutrition [26]. In the 2008 RAAB, the prevalence of known diabetes was 26.4% compared to 33.8% in our sample (increasing to 36.3% in Gaza) [12]. Accordingly, DR as a cause of blindness rose from 8.3% in 2008 to 24.1% in our sample overall and 32.4% in Gaza (the leading cause in the latter). No population data on diabetes in Gaza are available, but a study in the West Bank predicted diabetes prevalence of up to 21.5% of the population 25 years and older by 2030, highlighting high rates of obesity in both men and women and smoking among men as key risk factors [27]. Our estimates are higher, given the association between diabetes and age. Poor control among diabetic patients and limited availability of DR screening services is also of concern [28]; a clinic-based study in the West Bank estimated that only 21% of diabetic patients had controlled diabetes while over a third (37%) had never had an eye exam despite the same proportion experiencing retinopathy [29]. We did not capture DR barriers data in our survey, but evidence from elsewhere suggests that lack of physical access to health services due to protracted blockages, the lack of a national DR policy, limited availability of services and high cost of exams and treatments all contribute to low levels of DR screening among Palestinians [30–32].

In 2018–2019, cataract was the main cause of blindness overall (38.0%) and in the West Bank (51.4%), and second leading cause in Gaza (31.0%). This is a lower proportion of blindness compared to 2008 (55.0% overall, 56.1% West Bank, 54.0% Gaza), reflecting the increase in surgical output during this time period. In 2003, the cataract surgical rate (the number of surgeries per one million population per year) was estimated to be 843, increasing almost threefold to 2,117 by 2015 [33, 34]. However, while cataract surgical coverage and rates are higher, our findings show that good outcomes (i.e. eyes with a post-operative vision of 6/12 or better) were low across the oPt and particularly in Gaza, where the relative quality gap was above 50% at each of three surgical thresholds. Moreover, participants reported fear of surgery and lack of awareness as key barriers. Together, these results imply a need to focus on improving surgical quality rather than output or population access to services, for example through the use of biometry to inform lens power selection, alongside better monitoring of surgical outcomes and clear public health messaging [2, 24]. Efforts to improve the quality of available cataract surgical services should be prioritized post-crisis to decrease avoidable vision impairment across oPt.

As in the previous RAAB, adjusted prevalence of vision impairment and blindness was significantly higher in Gaza than in the West Bank. People in Gaza have historically had lower standards of living and more scarce health resources compared to the West Bank, in part due to limited movement of people and resources across the blockade [35].

The protracted conflict between Palestinian armed groups–mainly Hamas—and the Israeli military that began in October 2023 has monumentally impacted healthcare access, causing physical damage to infrastructure, lack of supplies and personnel and overwhelming emergency need, which have decimated routine access to care for chronic conditions or non-life-threatening impairments [36]. Service coverage is likely to have greatly or completely reduced in Gaza, increasing the backlog of required interventions to protect and restore sight. A complete rebuild of the health system may be necessary, ideally drawing on lessons learned from other post-conflict settings on development of responsive and equitable post-conflict health policies [37].

The survey results also highlight continued inequity between the sexes in accessing health services. The census-adjusted prevalence of any VI, blindness, diabetes and disability were all higher in women compared with men for oPt overall, as well as in each of the two zones, while cataract surgical coverage was lower for women compared with men at the <6/60 threshold, and effective cataract surgical coverage was lower for women compared with men at both the <6/18 and <6/12 thresholds. These persistent inequalities are in line with findings from elsewhere. A recent analysis of eCSC across 148 RAAB data sets from 55 countries determined a pooled risk difference of 3.2% for eCSC in women compared to men, highlighting lower access to quality cataract surgery for women overall [24]. Prevalence of both diabetes and disability is generally found to be higher in women than men, attributed to a mix of biological and social factors, including increased barriers to accessing health services [13, 15]. Important gaps between Palestinian men and women in health and access to health services exist. Women continue to face multiple difficulties in access to health facilities, believe that public health facilities do not have all the medical services they need, and believe that favoritism in medical referrals outside the public health sector exist [38].

Alongside the profound impact on routine health service provision and avoidable progression of disease, conflict exacerbates well-being issues for vulnerable population groups such as older people and people with disabilities [39]. Moreover, in the absence of well-functioning health and rehabilitative interventions, injuries caused by conflict can increase long-term disability in the population [40]. Prior to the conflict, we estimated that nearly a quarter of the population 50+ had a disability, similar to estimates for older people in other low and middle income settings that consistently show a strong association between disability and age [41]. This is likely to have increased on account of the conflict. Activities in pursuit of universal health coverage and healthy ageing must target equitable access to services for marginalized groups, including women and people with disabilities, to overcome such inequities.

Our study had many strengths. Response rate was high, minimizing non-response bias. Although the composition of the population was slightly different from the study participants, we presented sex-and age-adjusted estimates to allow for this. All teams received intensive training and were accompanied by the principal investigator frequently. Inter-observer variability was tested objectively by the RAAB trainer. In terms of limitations, the RAAB Trainer was not permitted to travel to Gaza to train the Gaza team and completed this training remotely while the PI facilitated the training in person. Further, our strategy of replacing households in which inhabitants were away may have introduced some selection bias, and our use of a rapid survey methodology may have under-estimated posterior segment disease.

In conclusion, our study found little difference in the prevalence of vision impairment and blindness in oPt compared with 2008, and emphasized the need for better quality cataract surgery and diabetic retinopathy services to decrease avoidable blindness across the oPt. Prevalence of both diabetes and disability was much higher than other estimates in the region, highlighting key areas for public health prioritization among older adults in oPt in the post-conflict era.

## Supporting information

S1 Checklist Inclusivity in global health research checklist. (DOCX)
